# Towards a personalized perspective on gliomas: an epigenetic-immuno-inflammatory aging framework

**DOI:** 10.3389/fimmu.2026.1898095

**Published:** 2026-07-22

**Authors:** Olga Vershinina, Victoria Turubanova, Claudio Franceschi, Mikhail Ivanchenko

**Affiliations:** 1 Research Center in Artificial Intelligence, Institute of Information Technologies, Mathematics and Mechanics, Lobachevsky State University, Nizhny Novgorod, Russia; 2 Institute of Biogerontology, Lobachevsky State University, Nizhny Novgorod, Russia; 3 Department of Genetics and Life Sciences, Sirius University, Sochi, Russia; 4 Institute of Neurosciences, Lobachevsky State University, Nizhny Novgorod, Russia

**Keywords:** age acceleration, aging, epigenetic clocks, glioma, inflammaging

## Abstract

High-grade gliomas cannot be fully understood without considering the biology of aging. In this study, we demonstrate that a set of genes previously established as predictors of tumor subtype and patient survival in gliomas can also be independently characterized as biomarkers associated with aging. Moreover, their expression changes during aging parallel with those seen in malignant transformation: genes which upregulation worsen prognosis are also upregulated with age. These findings support the hypothesis that glioma progression and aging share partially overlapping molecular mechanisms. We also demonstrate that while accelerated epigenetic and mitotic senescence – gauged by mathematical models (biological clocks) that track aging through specific changes in the DNA methylation of a set of CpG sites – appears inherent to various molecular variants of gliomas, its nature and prognostic significance vary depending on the specific subtype and the epigenetic clock model employed. Moreover, accelerated aging does not represent a universal hallmark of aggressiveness but rather a phenomenon specific to individual glioma subtype. In this paper, we provide a detailed discussion of the role of cellular senescence in glioma pathogenesis, with a particular focus on its key contribution to tumor growth control and therapeutic response. Additionally, we highlight the yet unresolved question of whether age-associated inflammation (inflammaging) acts as an independent driver of glioma progression or whether the tumor itself accelerates aging, and suggest the potential of profiling patients cellular aging in developing personalized treatment strategies.

## 1 Introduction

High-grade gliomas are the most aggressive primary tumors of the central nervous system. Gliomas are characterized by infiltrative growth, high proliferative activity, marked heterogeneity, and near-inevitable recurrence despite aggressive therapy, including resection, radiation, and temozolomide chemotherapy (1, 2). According to the 2021 WHO classification, high-grade gliomas encompass glioblastoma (grade 4), astrocytoma (grades 3 and 4), and oligodendroglioma (grade 3) (3). These tumor types differ markedly in their molecular profiles. Oligodendrogliomas carry IDH1/2 mutations coupled with complete 1p/19q codeletion, frequently accompanied by TERT promoter mutations and CIC/FUBP1 alterations. Astrocytomas share IDH1/2 mutations but lack 1p/19q codeletion and are defined by TP53 mutations and ATRX loss. Glioblastomas, which are predominantly IDH-wildtype, exhibit the most aggressive molecular phenotype, characterized by TERT promoter mutations, EGFR amplification, PTEN loss, and chromosome 7/10 alterations. The study (4) demonstrated that genes associated with epithelial-mesenchymal transition are more frequently altered in glioblastomas (GBM) than in lower-grade gliomas (LGG), reflecting a shift toward a more invasive phenotype. In terms of survival rate, for patients with glioblastoma, the most aggressive glioma subtype, median overall survival is approximately 12–15 months, and five-year survival does not surpass 5–10% (5).

The cellular origin of gliomas – especially glioblastoma – remains an open question, and the discussion in the literature continues. According to one of the most substantiated hypotheses, postnatal neural stem cells (NSCs) or their early progenitors, located predominantly in the subventricular zone (SVZ) and the dentate gyrus of the hippocampus, may serve as the cells of origin for glioblastomas. Lee et al. demonstrated that cells with an NSC phenotype harboring glioblastoma driver mutations are present in the human SVZ, leading the authors to identify this population as a likely tumor source (6). Subsequently, it has been established that NSCs can give rise to glioblastoma stem cells (GSCs), which, through complex interactions with the tumor microenvironment, play a decisive role in tumor growth, recurrence, and therapeutic resistance (7, 8). However, alongside this model, there is compelling evidence that gliomas can also originate from oligodendrocyte lineage progenitor cells, as well as through dedifferentiation of more mature glial cells driven by oncogenic mutations and microenvironmental signals (9). Under normal conditions, neural stem cells and progenitors that give rise to neuronal and glial lineages reside in the SVZ; however, in pathological states, mutations can accumulate in diverse cell populations, and the final tumor phenotype is shaped less by the cell of origin than by subsequent clonal evolution and transcriptomic plasticity (10, 11). It is important to note that during transformation and progression, GSCs can transition from a proneural to a mesenchymal phenotype, and their migratory capacity likely explains the emergence of malignant gliomas in various anatomical locations (6). Different tumor cell subpopulations mirror distinct developmental stages, forming – alongside genetic and epigenetic factors – the basis for glioblastoma heterogeneity (9). Consequently, despite extensive research, the primary cell of origin of gliomas remains unresolved, and the prevailing paradigm now regards the SVZ as a niche facilitating the accumulation of mutant cells rather than the exclusive and obligatory source of tumor initiation.

Aging significantly contributes to cancer development and progression. This link is underpinned by shared biological hallmarks, among them genomic instability, increased mutational burden, aberrant telomeres, epigenetic alterations, inflammation, immune injury, metabolic reprogramming, and degradation system impairment (12). Aging is accompanied by declining immune function and chronic low-grade inflammation, which together increase cancer susceptibility in older adults (13, 14). In particular, the incidence of glioblastoma rises sharply with age, peaking at 60–70 years, in contrast to lower-grade gliomas (15). Meanwhile, patients of the same age with identical tumor histopathology may exhibit markedly different treatment responses and survival outcomes due to the molecular heterogeneity of gliomas. This hidden variability underscores the need for in-depth transcriptomic and epigenomic profiling of tumors, which enables the identification of distinct molecular subtypes and provides more accurate personalized prognostic information (16). Currently, MGMT promoter methylation is among the most informative epigenetic markers of therapeutic response. Yet, the analysis of individual locus often fails to capture the complex systemic changes occurring in tumor tissue. Consequently, integrative measures such as tumor epigenetic age – a personalized estimate of tissue aging based on DNA methylation, referred to as the epigenetic clock – are gaining increasing attention. Prior studies have demonstrated that gliomas display accelerated epigenetic aging relative to normal brain tissue (17). Whether epigenetic age acceleration or deceleration is associated with patient survival remains an open question, as existing data are contradictory (18–21). It remains unclear whether the relationship between epigenetic age acceleration and prognosis is universal across all glioma types (glioblastoma, astrocytoma, and oligodendroglioma) or varies by molecular subtype.

The internal molecular mechanisms linking cellular senescence to tumor aggressiveness also remain poorly understood. Previous studies have shown that glioblastoma can suppress cellular senescence by upregulating VEGFR2 expression, thereby maintaining its invasiveness (22). There are suggestions that cellular senescence drives the deep invasion of residual glioblastoma cells into the brain parenchyma (23). The senescence-associated secretory phenotype (SASP) plays a pivotal role in glioma pathogenesis. Although cellular senescence is traditionally regarded as a tumor-suppressive mechanism, in high-grade gliomas SASP functions as a key driver of tumor progression (24). Induced by chemotherapy and radiation therapy, SASP paradoxically remodels the brain microenvironment, promoting glioma cell stemness, angiogenesis, and immune evasion, thereby emerging as a critical target for overcoming therapeutic resistance and preventing relapse. In this regard, developing specialized methods to assess the rate of aging at the cellular level could offer a novel approach for predicting treatment efficacy and patient survival.

Furthermore, age is recognized as one of the most important clinical factors influencing treatment response in glioma patients (25–27). Older patients consistently show worse response rates and reduced survival, regardless of the treatment modality. Of particular interest is the potential interplay between age and response to immunotherapy – a treatment whose efficacy in gliomas remains limited, partly owing to the “cold” tumor microenvironment with scarce CD8+ T cell infiltration. A recent meta-analysis (28) has shown that age may influence survival in glioblastoma immunotherapy; however, this remains an open question, largely due to the bias toward recruiting younger and healthier patients into clinical trials.

This study aims to address the above-mentioned questions regarding the interplay between aging – especially epigenetic aging – and glioma prognosis, as well as to discuss the potential relationship between the efficacy of immunotherapy in gliomas and age-associated alterations in the tumor microenvironment.

## 2 Association of glioma subtype- and survival-related genes with age

Aggressive gliomas show a pronounced age-associated rise in incidence, with a peak between 60 and 70 years of age, yet the molecular mechanisms linking the aging process to glioma malignancy remain incompletely understood (15). In the study (29), the authors used a syngeneic murine glioma model to demonstrate that aging is associated with changes in the brain environment that promote more aggressive glioma infiltration. These changes are also characterized by increased glial reactivity and diminished functional resilience to tumor burden. In the examined mouse model, older animals exhibited worse survival than younger ones, despite identical tumor implantation and postoperative care. These findings underscore the importance of considering aging as a critical factor in glioma research.

A growing body of evidence indicates that genes governing the aging process can serve powerful prognostic markers in glioma patients (30–33). In these studies, the authors identified aging-associated gene sets from the literature or public databases and then, through bioinformatics analysis, showed that their expression can serve as prognostic biomarkers for glioma. This raising recognition of shared aging–cancer markers motivated us to re-examine the gene signature that we previously developed for predicting histological glioma subtype and survival (34). Specifically, we reversed the process and investigated whether the 13 genes (TERT, NOX4, MMP9, TRIM67, ZDHHC18, HDAC1, TUBB6, ADM, NOG, CHEK2, KCNJ11, KCNIP2, and VEGFA), originally selected for their prognostic significance in gliomas, are also involved in the aging process. As we previously demonstrated, the expression of these genes is associated with survival outcomes in patients with glioma (34). In particular, increased expression of TERT, NOX4, MMP9, ZDHHC18, HDAC1, TUBB6, ADM, CHEK2, and VEGFA was associated with decreased survival (hazard ratio (HR) > 1), whereas increased expression of TRIM67, NOG, KCNJ11, and KCNIP2 was associated with increased survival (HR < 1). Specific HR values for gliomas from The Cancer Genome Atlas (TCGA) are presented in **Supplementary Table 1**. Using the same RNA-seq data here, we calculated Spearman correlation coefficients to assess age-related expression changes and found comparable patterns (**Supplementary Table 1**): expression was positively correlated with age for TERT, NOX4, MMP9, ZDHHC18, HDAC1, TUBB6, ADM, CHEK2, and VEGFA, and negatively correlated with age for TRIM67, NOG, KCNJ11, and KCNIP2 (Benjamini−Hochberg (BH) adjusted p-value (padj) < 0.05). Furthermore, age per se constitutes an important variable for survival prediction (HR > 1).

We then analyzed the scientific literature and, while we found no mention of these genes at the intersection of gliomas and aging, we did find compelling evidence for their involvement in the aging process in general. This is consistent with the notion that glioma carcinogenesis and aging may share common features. It emerged that the telomerase reverse transcriptase (TERT) plays a key role in aging by preserving telomere length, a critical biomarker of cellular senescence. However, in all tissues, the TERT gene is epigenetically repressed during aging. This silencing leads to telomere dysfunction, which in turn drives the molecular and cellular damage underlying both the aging process and age-related diseases (35). It has been suggested that NADPH oxidases of the NOX family act as professional generators of reactive oxygen species and may play a significant role in the aging process and age-related diseases (36). For example, NOX4 expression has been shown to be increased in the vessels and mitochondria of old mice, contributing to the development of cardiovascular disease with aging (37). The role of matrix metalloproteinases (MMPs) in aging has been discussed in detail (38). These enzymes are involved in extracellular matrix remodeling, a process that undergoes numerous changes with age. Specifically, another study showed that serum MMP9 levels increase significantly with age (39). The histone deacetylase 1 (HDAC1) expression in the periventricular white matter was found to be 31% higher in elderly donors compared to young donors (40). This observation aligns with subsequent work showing that HDAC1 become elevated with age in microglia across both mouse and human tissues (41). Angiogenic and vascular factors further illustrate the aging-associated shift. In a hippocampal gene expression study, VEGFA levels were elevated in aged adults compared to younger individuals (42). Moreover, at the protein level, plasma VEGFA is a component of several inflammatory aging clocks and has been shown to positively correlate with age (43–46). A similar age-related increase has been observed for adrenomedullin (ADM): protein extracts from the frontal cortex of healthy older adults contained significantly higher ADM levels than those from younger donors (47), and plasma ADM levels also increased with age (48). In contrast, some genes with neuroprotective roles appear to decline with age. For instance, aged patients with traumatic brain injury (TBI) exhibited reduced blood-based expression of the noggin (NOG) gene, a factor linked to neurorecovery and neuroregeneration, compared to younger patients, with these differences becoming even more pronounced one week after injury (49). Older age is known to be an independent factor in poorer recovery after TBI, an effect associated with age-related changes in neuroinflammation (50). Thus, the concordant direction of age-related NOG expression changes in TBI and glioma may suggest shared molecular mechanisms of aging that modulate both responses to brain injury and tumor progression. For the remaining markers, associations with aging have either not yet been detected or are limited to animal data. In mice, Zdhhc18 expression in the spinal cord was found to increase with age (51). Similarly, Tubb6 expression was elevated in aging mice and has been implicated in neurodegenerative disease processes (52). Finally, hippocampal Kcnip2 expression in rats was reduced in the “elderly without cognitive impairment” group relative to both the “young” and “elderly with cognitive impairment” groups, suggesting a potential role for this gene in maintaining cognitive function during aging (53).

Collectively, the evidence reviewed here demonstrates the existence of a subset of glioma prognostic biomarkers that also function as molecular indicators of aging. For the genes examined, the direction of association is consistent: expression changes predictive of worse survival in glioma mirror aging. This observed overlap, while based on reviewed studies, lends support to the concept that glioma progression and aging engage partially shared molecular mechanisms, and the identified biomarkers may serve as potential targets for future therapeutic investigation.

## 3 Epigenetic aging in gliomas

Natural aging induces epigenetic shifts and gene expression changes in the central nervous system. DNA methylation, a key epigenetic mechanism of gene regulation, is constantly altered during both normal aging and malignant transformation. With age, normal tissues undergo global hypomethylation alongside focal hypermethylation at CpG islands in promoters of genes linked to aging and cell cycle control. In cancer, including gliomas, these age-related changes are exacerbated and reprogrammed, producing tumor-specific aberrant methylation patterns that affect DNA repair genes (e.g., MGMT), tumor suppressors, and genomic repeats. MGMT encodes a DNA repair enzyme that counteracts alkylating agents such as temozolomide. Promoter methylation silences MGMT, impairs its repair function, and thereby enhances chemosensitivity. Accordingly, patients with IDH-mutant tumors or methylated MGMT promoters generally manifest longer survival (5, 54, 55), whereas recurrent glioblastoma exhibits a median survival of only six months. Thus, DNA methylation represents a shared mechanism between aging and carcinogenesis, and its analysis enables the evaluation of both tissue biological age and tumor prognostic features (56).

To date, numerous epigenetic clock models have been developed to predict biological age based on the methylation status of CpG sites (57–60). It is well established that accelerated epigenetic aging, whereby predicted biological age exceeds chronological age, is associated with an elevated risk of developing various diseases, including cancer, and also constitutes a biomarker of increased mortality (61). In gliomas, it has been shown that tumors exhibit accelerated epigenetic and mitotic aging compared to normal brain tissue, as measured by the Horvath and epiTOC clocks (18). The authors also reported a possible non−trivial association between accelerated aging (measured by these clocks) and improved survival in a mixed glioma sample not separated by subtype. Another study also reported that accelerated epigenetic aging, as measured by the Horvath clock, positively impacted overall survival in glioma patients (19). However, after subdividing gliomas into subtypes, the statistically significant association (unadjusted p-value (p) = 0.011) remained only for the Classic-like subtype. In paper (20), age acceleration based on the Horvath clock, observed in IDH wild-type glioblastoma, was also associated with better outcomes. To determine whether this result is attributable to the Horvath clock or to the complex biology of glioblastomas, and whether the association of accelerated aging with survival is consistent across glioma subtypes, we analyzed multiple biological aging clocks in patients from The Cancer Genome Atlas (TCGA) (6).

The analysis drew upon Illumina HumanMethylation450 BeadChip DNA methylation data (beta values) for solid tumors, sourced from the TCGA-LGG and TCGA-GBM datasets, and an extended set of clinical annotations from the cBioPortal. Patients were divided into three molecular glioma subtypes based on IDH and 1p/19q co-deletion status: glioblastoma (IDH-wt, n = 210), astrocytoma (IDH-mut-noncodel, n = 253), and oligodendroglioma (IDH-mut-codel, n = 167). A control group of normal brain frontal lobe samples (n = 29) was collected from the GSE41826 (62), GSE40360 (63), GSE53162 (64), and GSE59457 (65) datasets. The data preprocessing pipeline included all required steps: filtering, imputation, normalization, and batch correction. To comprehensively evaluate biological aging, 24 different epigenetic models were employed. First, multi-tissue clocks Horvath (57), PCHorvath (59), AltumAge (66), SkinAndBlood (67), and PCSkinAndBlood (59), as well as the brain aging clock DNAmClockCortical (68), were considered. Secondly, some blood-based clocks were used, such as Hannum (58), PCHannum (59), GrimAge (69), PCGrimAge (59), GrimAge2 (70), DNAmPhenoAge (71), PCPhenoAge (59), Lin (72), YingCausAge (73), StocH, StocP, StocZ (74), and EpInflammAge (46). Additionally, the mitotic clocks epiTOC (75), stemTOC (76), and RepliTali (77), which reflect different aspects of cellular proliferative activity, and the telomere clocks DNAmTL (78) and PCDNAmTL (59) were examined. The DNAmClockCortical and EpInflammAge clocks were obtained from the repositories published by the authors of the corresponding studies, while the remaining clocks were obtained from Python’s pyaging package (79).

**Figure 1:**
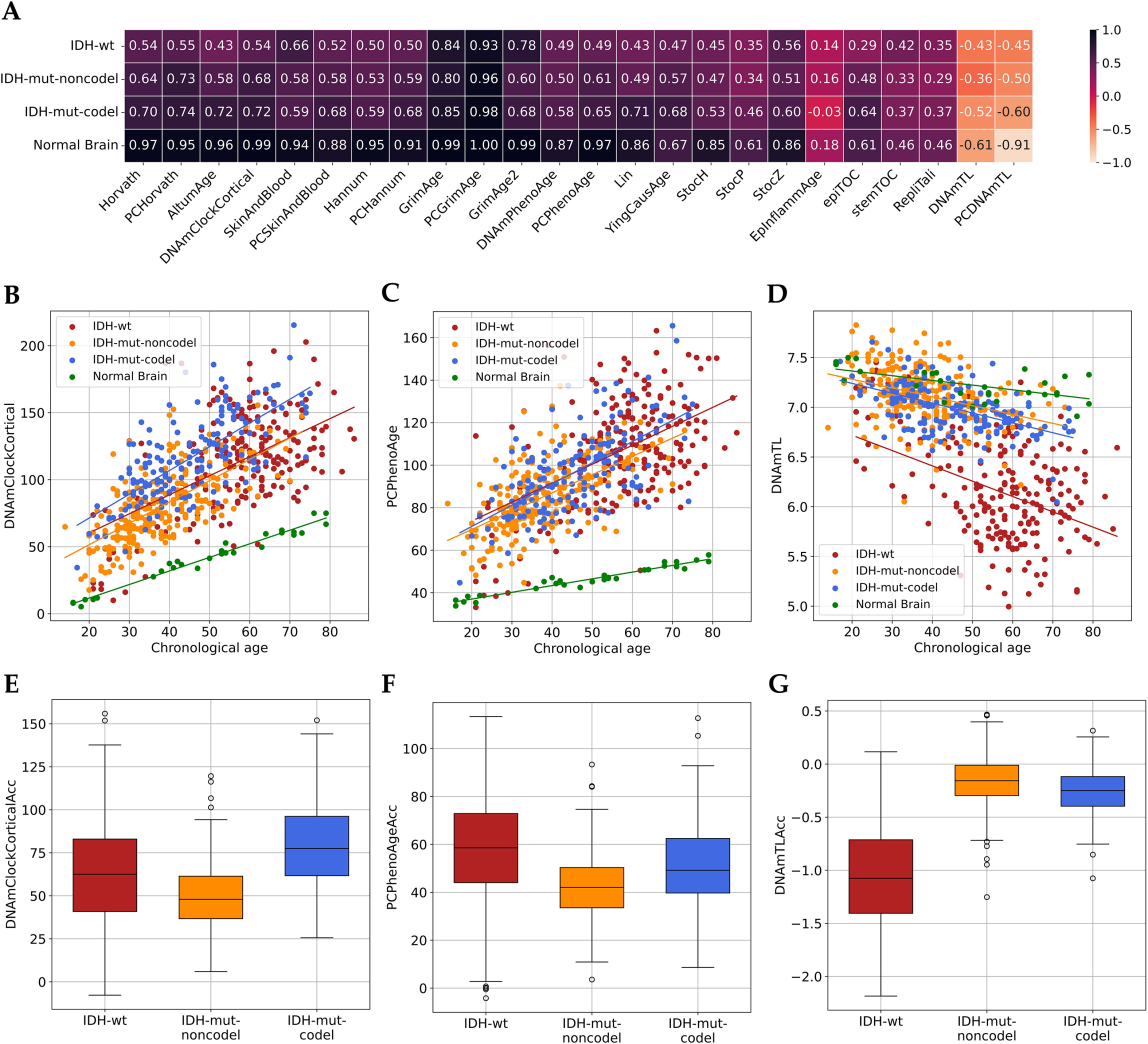
Relationship between chronological age and epigenetic measures of aging. **(A)** Pearson correlation of each aging biomarker (epigenetic age, mitotic age, and telomere length) with chronological age. **(B)** DNAmClockCortical age, **(C)** PCPhenoAge, and **(D)** DNAmTL versus chronological age in glioma IDH-mutation–1p/19q codeletion subtypes and normal brain. **(E, F)** Epigenetic age acceleration (residuals from the normal brain regression) across glioma subtypes for **(E)** DNAmClockCortical and **(F)** PCPhenoAge clocks. **(G)** DNAmTL-based telomere length attrition in glioma subtypes relative to normal brain.

First, we assessed the correlation between predicted values and chronological age using Pearson’s coefficient (**Figure 1A**). In both healthy brain tissue and glioma subtypes, epigenetic and mitotic clocks showed a positive correlation with chronological age. In contrast, telomere length estimates were negatively correlated with age. The magnitude of the correlation coefficient decreased from normal brain tissue to gliomas, with glioblastoma showing the weakest correlation among all subtypes. The GrimAge and PCGrimAge clocks yielded the highest correlations across all four sample groups (r > 0.80). The lowest coefficients were obtained for EpInflammAge (r < 0.18), possibly rooted in the poor applicability of the epigenome-inflammatory clock – trained on blood inflammation markers – to brain tissue. It was concordant with the documented difference of inflammatory signatures between blood and brain (80). Therefore, it was excluded from further consideration. Predictions of several clocks are presented in **Figures 1B–D**. Notably, the multi-tissue clock achieves strong predictive accuracy in the control sample (mean absolute error (MAE): 4–8 years). The blood clock tends to underestimate predictions and shows greater variability, although remaining highly correlated with age (MAE: 6.5–68 years).

Next, epigenetic age acceleration (for epigenetic clocks that produce the output age in years), mitotic age acceleration (for epiTOC, stemTOC, and RepliTali), and telomere length attrition (for DNAmTL and PCDNAmTL) were each defined as the residual from a linear regression of the respective clock output on chronological age, fitted in the normal reference cohort. For brevity, we refer to all these residuals collectively as age acceleration (Acc) throughout the manuscript, with the understanding that for DNAmTL and PCDNAmTL, negative values reflect telomere shortening relative to the age-matched normal brain. Deviations of age acceleration from zero – the expected mean of the normal reference cohort – were assessed using the Wilcoxon signed-rank test. The analysis revealed that each glioma subtype exhibits accelerated epigenetic and mitotic aging across virtually all examined clocks (padj < 0.0001). DNAm-predicted telomere length was reduced in gliomas compared to age-matched healthy brain (padj < 0.0001), with the most pronounced shortening observed in glioblastomas.We assessed differences in accelerated aging among the three glioma subtypes using the Kruskal−Wallis test with Dunn’s *post hoc* test. Among the three subtypes, oligodendrogliomas showed the greatest acceleration of epigenetic age as measured by the Horvath, PCHorvath, AltumAge, DNAmClockCortical, SkinAndBlood, and Hannum clocks. The mitotic clocks epiTOC, stemTOC, and RepliTali were also more accelerated in oligodendrogliomas. In contrast, the remaining 12 epigenetic clocks demonstrated the greatest age acceleration in IDHwt glioblastomas. Additionally, telomere length attrition, as assessed by the DNAmTL and PCDNAmTL clocks, was most pronounced in glioblastomas. **Figures 1E–G** show examples of Acc for several clocks.

**Figure 2:**
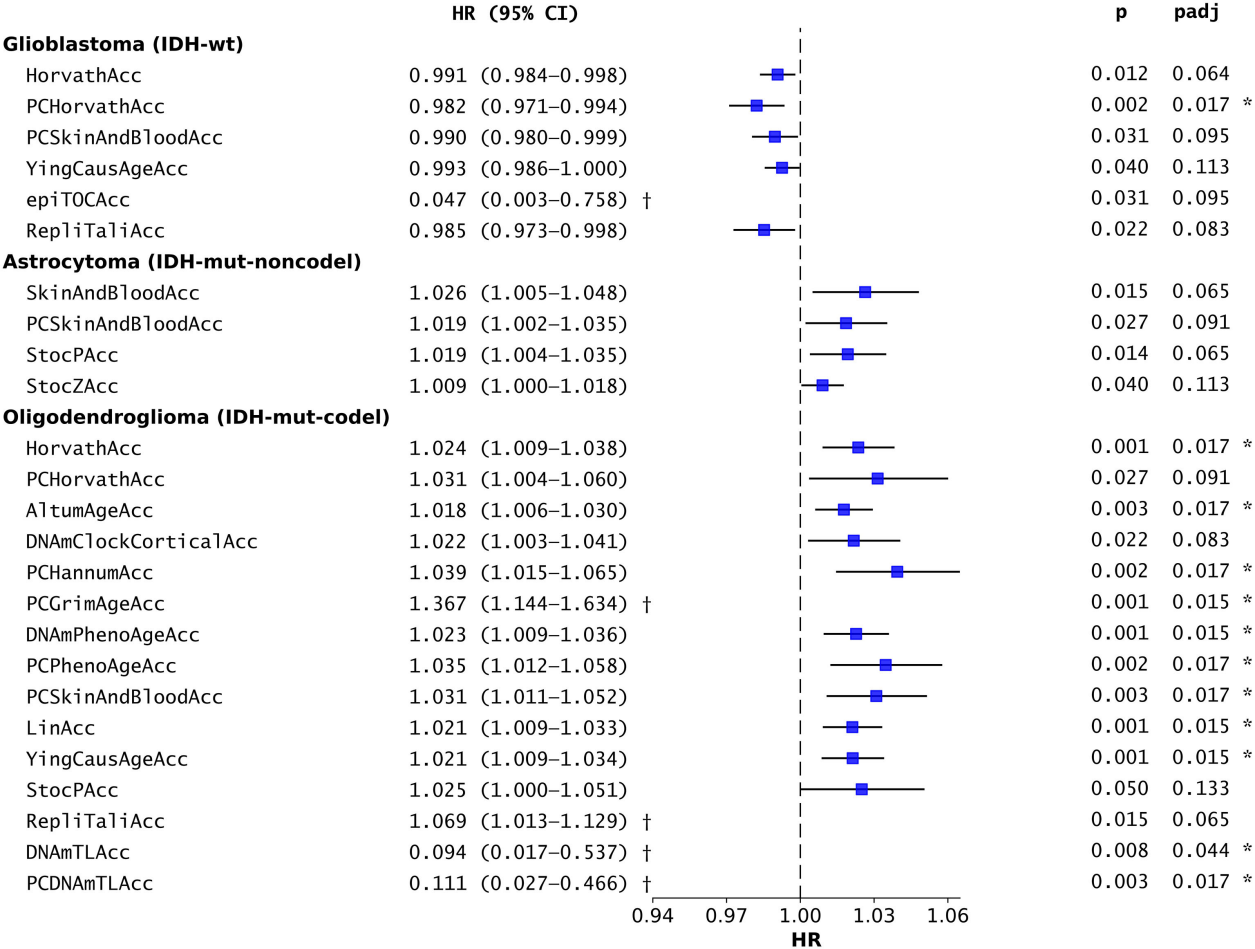
Forest plot shows the association of age acceleration (Acc) with patient overall survival in each subtype. Only clocks with p < 0.05 are shown. † HR values falling outside the axis limits [0.94, 1.06] were omitted from the forest plot. *padj < 0.05, BH-corrected.

For each clock, the association between age acceleration and overall survival in glioma patients was assessed using a stratified Cox proportional hazards model that included an interaction between acceleration and glioma subtype. Model was adjusted for sex and tumor grade. Stratification was performed by chronological age groups (<40, 40–60, >60 years) and MGMT promoter status because these variables violated the proportional hazards assumption in glioblastomas (Schoenfeld test, p > 0.05). Hazard ratios for each subtype were obtained by running the model three times, each with the desired reference category. **Figure 2** shows statistically significant results. After BH correction, Acc measured by PCHorvath clock was significantly associated with survival in glioblastoma, with greater acceleration linked to longer survival (HR < 1, padj < 0.05). Notably, prior to correction, five clocks – Horvath, PCSkinAndBlood, YingCausAge, epiTOC, and RepliTali – also showed an association in the same direction (p < 0.05). A nominal association was observed in astrocytomas, where increased acceleration across four clocks (SkinAndBlood, PCSkinAndBlood, StocP, and StocZ) was linked to decreased survival (HR > 1); however, this finding did not remain significant after correction. In oligodendrogliomas, acceleration of epigenetic age was consistently associated with decreased survival, as confirmed by nine epigenetic clocks: Horvath, AltumAge, PCHannum, PCGrimAge, DNAmPhenoAge, PCPhenoAge, PCSkinAndBlood, Lin, and YingCausAge (padj < 0.05). Associations for the PCHorvath, DNAmClockCortical, StocP, and RepliTali clocks did not withstand BH correction. Furthermore, telomere shortening, quantified by the DNAmTL and PCDNAmTL clocks, was also found to be associated with decreased survival in oligodendrogliomas (HR < 1, padj < 0.05).

We then conducted a similar analysis of the association between age acceleration and survival, taking into account methylation subtypes (16). The distribution of patients from TCGA by these subtype was as follows: IDHwt/Mesenchymal-like (n = 99), IDHwt/Classic-like (n = 73), IDHwt/PA-like (n = 26), IDHwt/LGm6-GBM (n = 12), IDHmut-non-codel/G-CIMP-high (n = 238), IDHmut-non-codel/G-CIMP-low (n = 11), IDHmut-non-codel/Codel (n = 4), and IDHmut-codel/Codel (n = 167). Subtypes containing fewer than 20 samples were excluded from further survival analysis to avoid unstable coefficient estimates. After BH correction, statistically significant associations remained only for IDHmut-codel/Codel subtype. Specifically, nine epigenetic clocks (where greater acceleration was associated with shorter survival) and the PCDNAmTL telomere length clock (where shorter telomeres were associated with shorter survival) reached significance (padj < 0.05). In terms of nominal significance, only one result was obtained for glioblastoma (p = 0.045): in IDHwt/PA-like tumors, which lack canonical telomere maintenance mechanisms (TERT mutations and ATRX loss), telomere shortening assessed by PCDNAmTL clock was associated with reduced survival.

Thus, the previously reported protective effect of epigenetic age acceleration in glioblastoma is likely a result of pooling methylation data for subtypes that differ in both global DNA methylation levels and clinical course. When IDHwt glioblastomas are stratified into groups with homogeneous methylation patterns – Mesenchymal-like, Classic-like, and PA-like – this paradoxical signal disappears entirely. Still, validation in independent, larger cohorts remains due.

## 4 From firefighter to arsonist: the dual role of SASP in cancer

The senescence-associated secretory phenotype (SASP) refers to a paracrine signal released by senescent cells and constitutes a characteristically proinflammatory secretome. However, emerging evidence suggests that this fundamental physiological mechanism can be co-opted by cancer cells to promote their evolution and immune evasion. Although senescent cells undergo proliferative arrest, the SASP signals they secrete can actively support cancer progression by reprogramming residual malignant cells and non-cancerous components of the tumor microenvironment toward pro-tumorigenic phenotypes (24, 81). Furthermore, paracrine signals and immunosuppressive cytokines released by senescent malignant and non-cancerous cells contribute to the establishment of an immunosuppressive tumor microenvironment and represent a potential mechanism of resistance to immunotherapy (82).

Senescent glioma cells secrete a broad array of proinflammatory cytokines and chemokines, notably IL-6, IL-8, and CXCL1. These cytokines activate the NF-κB and STAT3 signaling pathways in recipient tumor and stromal cells, driving survival, proliferation, and resistance to apoptosis. The chronic inflammation sustained by SASP perpetuates a vicious cycle: it induces oxidative stress and DNA damage in neighboring non-senescent cells, thereby increasing genomic instability and accelerating their malignant transformation (83). Furthermore, SASP encompasses the secretion of proangiogenic factors such as VEGF and PlGF. These factors activate perivascular cells and the endothelium, driving neoangiogenesis—the formation of a chaotic, tortuous, and hyperpermeable vascular network. Angiogenesis is critical in glioblastoma: this aberrant hypervascularization supplies the rapidly growing tumor with oxygen and nutrients, facilitates its escape from hypoxic constraints, and contributes to the tumor’s aggressive infiltration of the brain parenchyma. Moreover, hypoxia itself amplifies the SASP secretory phenotype and stabilizes HIF-1α, creating a positive feedback loop that further stimulates the secretion of VEGF and proinflammatory cytokines, thereby progressively worsening the prognosis.

A more pronounced senescent phenotype correlates with the grade of glioma malignancy. For instance, Al Shboul et al. (84) demonstrated that glioblastoma relapse is accompanied by a transcriptional shift toward a senescence-associated state, without concomitant changes in SOX2 or immune markers. In aging gliomas, SOX2 functions as a critical transcription factor that sustains the stemness, treatment resistance, and plasticity of cancer stem cells. While baseline SOX2 expression naturally declines with physiological aging in normal neural stem cells, its aberrant overexpression in gliomas actively bypasses cellular senescence to drive tumor growth. Additionally, a study by Tan et al. (81) showed that cellular senescence-associated genes, such as PTTG1 and MYC, significantly influence glioma cell activity.

The current understanding of cellular senescence recognizes this state not as a single, fixed phenotype but as a spectrum of adaptive responses, the biological consequences of which depend on the nature of the initiating insult. Five major subtypes are distinguished based on their molecular mechanisms of initiation. The first group comprises endogenous, chronologically driven mechanisms: replicative senescence (RS), caused by critical telomere shortening and activation of the ATM–p53/p21 cascade, and mitochondrial dysfunction-associated senescence (MiDAS), which arises from redox imbalance and a decline in the NAD^+^ pool, triggering an AMPK-dependent response via the LKB1–p53 axis, typically without a requirement for substantial nuclear DNA damage. The second group consists of stress-induced subtypes driven primarily by genotoxic or replication stress: oncogene-induced senescence (OIS), which paradoxically results from hyperactivation of proto-oncogenes such as RAS or BRAF and is largely mediated through the p16/INK4a–Rb pathway, and therapy-induced senescence (TIS), a consequence of chemo- or radiotherapy that causes extensive DNA damage, often accompanied by the formation of polyploid and giant cells. Finally, a distinct category is immunologically induced senescence (IIS), which operates via a paracrine mechanism: proinflammatory cytokines such as IFN-γ and TNF-α activate JAK/STAT signaling and, independently of telomere status, induce p21/WAF1 expression, thereby converting an inflammatory signal into a cell cycle arrest phenotype (85, 86).

A critical consequence of this heterogeneity lies in the distinct secretory profiles that define both the physiological role and pathogenic potential of each senescence subtype. RS is associated with a relatively mild, chronic SASP that contributes to age-related tissue homeostasis, whereas OIS and TIS exhibit an aggressive proinflammatory and proangiogenic phenotype – characterized by hypersecretion of IL-6, IL-8, VEGF, and matrix metalloproteinases – that, in an oncological context, fosters a microenvironment conducive to relapse and metastasis. MiDAS, by contrast, displays a qualitatively distinct SASP profile, often marked by a predominance of IL-8 without concomitant overexpression of IL-6, suggesting metabolically driven regulation of the secretome. The IIS-associated SASP, in turn, is enriched in interferon-induced chemokines such as CXCL10 and CCL2 and functions less as a reflection of intrinsic cellular instability than as a signaling mechanism that propagates senescence to neighboring cells during chronic inflammation.

The connection between SASP and immune-inflammatory events is mediated by the production of proinflammatory cytokines and the activation of signaling cascades, including NF-κB (partly via the cGAS-STING axis), which initiate a self-perpetuating inflammatory cycle. This drives the development of chronic low-grade inflammation. In addition, SASP induces paracrine senescence in neighboring cells and promotes senescence of immune cells, which further amplifies the secretion of proinflammatory factors and attenuates immune surveillance, thereby closing a vicious circle (87–89).

The paradoxical role of SASP in carcinogenesis is governed by the temporal context. In an acute setting, SASP exerts tumor-suppressive functions: secreted chemokines recruit effector immune cells (macrophages, NK cells, and T lymphocytes) to eliminate senescent and transformed cells. However, upon chronic persistence of senescent cells-driven by age-related immune system decline SASP acquires pro-oncogenic properties. Sustained secretion of IL-6 and TNF-α perpetuates mutagenic inflammation, while recruitment of myeloid-derived suppressor cells and regulatory T cells (Tregs) suppresses antitumor immunity. Concurrently, matrix metalloproteinases (MMPs) facilitate invasion, and VEGF promotes angiogenesis, collectively establishing a microenvironment favorable for tumor growth and metastasis. This functional switch from elimination to tumor protection constitutes the core paradox of SASP.

## 5 The role of inflammaging in modulating the response to glioma immunotherapy

Therapy for gliomas presents a complex clinical challenge due to the combination of several interrelated factors. These include pronounced intratumoral heterogeneity, the restoration of tumor potential via stem cell components (including neural stem cells), difficulties in early diagnosis due to challenging tumor localization, aggressive invasion into the brain parenchyma, and the formation of an immunosuppressive “cold” microenvironment (90, 91). These features significantly limit the efficacy of standard therapy. However, modern immunotherapeutic strategies can overcome some of these barriers.

Approaches such as immune checkpoint inhibitors, CAR-T cells, and antitumor vaccines demonstrate limited and variable efficacy against gliomas, largely due to the high heterogeneity of patient responses (92, 93). In this context, the patient’s biological age-specifically, the rate of organismal aging, which does not always correlate with chronological age-emerges as an underevaluated factor. With respect to glioma pathogenesis, it is essential to distinguish between systemic aging of the entire organism and local aging of the tumor microenvironment, as each of these processes independently contributes to resistance to immunotherapy.

One of the most significant challenges limiting the efficacy of glioma immunotherapy is the immunosuppressive tumor microenvironment. The brain is characterized by a substantially reduced T-cell population compared to other organs, and the glioblastoma microenvironment consists predominantly of macrophages, which in proximity to tumor cells often perform protumorigenic functions. Among the key components of the TME contributing to immunosuppression are TGF-β2, PGE-2, IL-6, and IL-10 (94, 95).

Inflammaging refers to the chronic, low-grade inflammation that naturally accompanies aging. In the context of high-grade malignant gliomas, progressive immune dysfunction accelerates tumor progression. Age-related inflammatory processes, including elevated cytokine levels, create a favorable immunosuppressive microenvironment that actively promotes rapid glioma cell proliferation and increases tumor “coldness”. Under the influence of a chronic cytokine profile (IL-6, TNF-α), resident microglia and macrophages switch to a protumorigenic state (a phenotype resembling M2). Instead of fighting the glioma, they begin to stimulate its growth and angiogenesis (96).

The checkpoint protein PD-L1 is elevated in glioblastoma, which is associated with poor prognosis due to tumor evasion of the immune response. Inflammaging acts as a direct trigger for hyperactivation of the PD-1/PD-L1 axis, ultimately “freezing” the antitumor immune response. PD-L1 expression positively correlates with the level of immunosuppressive cells in the tumor microenvironment and negatively correlates with the level of cytotoxic immune cells. High PD-L1 expression also significantly correlates with the transition of tumor-associated macrophages to an anti-inflammatory (M2) phase. Peripherally, PD-L1 expression is associated with an increased proportion of regulatory T cells in glioblastoma.

Systemic immunosuppression can be pronounced in patients with glioblastoma, representing a significant barrier to the success of immunotherapy. The number of CD4^+^ T cells in patients with glioblastoma can reach levels comparable to those in patients with acquired immunodeficiency syndrome. Furthermore, glioma cells express elevated levels of the ligand PD-L1 and indoleamine-2,3-dioxygenase (IDO), thereby limiting antigen presentation (97).

Biomarkers have been reported that may overcome the limitations of traditional indicators of therapeutic response in glioblastoma (98). For instance, ADAMTSL4, whose high expression level is associated with an immunosuppressive microenvironment and worse overall survival. ADAMTSL4 correlates with immune checkpoints (PD-1, PD-L1, CTLA-4) and stromal infiltration. The marker ACSS3 predicts resistance to immune checkpoint inhibitor therapy. *In vitro* studies have demonstrated that CD81 promotes proliferation, migration, and stemness properties of glioblastoma cells while inhibiting apoptosis, confirming its potential as a novel immune-dependent biomarker.

Meanwhile, a fundamental question remains unresolved: whether age-associated inflammation (inflammaging) in patients with glioma acts as an independent driver of disease progression, or whether the tumor itself accelerates local and systemic aging, thereby creating a vicious circle. Resolving this dilemma has direct therapeutic implications. In the first scenario, the application of gerontological interventions – senolytics or anti-inflammatory strategies – is justified. In the second scenario, early aggressive cytoreductive treatment takes on priority significance. Until this question receives empirical resolution, any attempts to predict response to immunotherapy based on biomarkers will remain purely descriptive rather than causal in nature.

## 6 Discussion

Chronological age is one of the most robust prognostic factors in glioma, yet the mechanisms underlying this association and the role of biological aging remain poorly understood. Although glioma can arise at any age, the clinical course of the disease changes with advancing age, and the risk of developing glioblastoma – the most aggressive form – rises sharply. Numerous studies have confirmed that patient age at diagnosis serves as an independent prognostic factor: even within the same histological group, prognosis can vary substantially with age, and survival steadily declines as age at diagnosis increases. However, it remains unclear whether accelerated or decelerated biological aging influences survival and whether deviations in biological age can be used as predictors of disease progression. Gliomas and aging share several fundamental mechanisms, including genomic instability, telomere shortening, and epigenetic alterations. Moreover, specific molecular and epigenetic markers highlight the link between tumor progression and biological aging, and may therefore serve as valuable prognostic tools.

Different molecular subtypes of glioma exhibit distinct patterns of epigenetic aging. It has previously been shown that IDH-mutant, 1p/19q-codeleted gliomas, despite their more favorable prognosis, paradoxically exhibit greater epigenetic aging as measured by the Horvath clock than IDH-wildtype glioblastomas (18). Here, having evaluated 24 different DNA methylation-based epigenetic clock models, we found that oligodendrogliomas (IDH-mut-codel) indeed exhibited significant age acceleration according to multi-tissue clocks (Horvath, PCHorvath, AltumAge, SkinAndBlood), the brain-specific clock (DNAmClockCortical), the Hannum clock, and the mitotic clocks. This phenomenon likely stems from IDH-induced global hypermethylation (99), which probably extends to the age-associated CpG sites within a given clock. In the Horvath model, for instance, methylation levels increase with age at more than half of the constituent CpGs (57), a bias that may lead to the overestimation of epigenetic age in IDH-mutant oligodendrogliomas. The greatest mitotic clock acceleration likely reflects the long subclinical history of IDH-mutant gliomas. Unlike *de novo* glioblastomas, these tumors can progress for years before diagnosis, accumulating a far greater lifetime number of cell divisions. However, establishing the causes of accelerated aging in oligodendrogliomas will require detailed examination of the individual clock CpG sites and their methylation patterns across glioma subtypes. Glioblastomas showed significant age acceleration according to 12 clocks that capture mortality risk (GrimAge), phenotypic aging (DNAmPhenoAge), and stochastic epigenetic variation (StocH, StocP, StocZ), alongside the most pronounced telomere attrition (DNAmTL, PCDNAmTL). The acceleration of clocks tracking genuine physiological deterioration – inflammation, immune and tissue dysfunction, and replicative wear – suggests an aging pattern directly aligned with the aggressiveness of glioblastomas.

Regarding the association between accelerated aging and survival, previous findings from predominantly IDH-wildtype cohorts indicate that greater epigenetic age acceleration in tumors is associated with improved survival (18–20). These observations contrast the data obtained from normal tissues, where epigenetic age acceleration consistently correlates with greater morbidity and mortality. In our analysis of 24 clock models, we found that the protective effect of age acceleration in IDH-wildtype glioblastoma was reproduce by the PCHorvath clock only. However, stratification of these tumors into molecularly homogeneous methylation subtypes (Mesenchymal-like, Classic-like, and PA-like) removed this signal, rendering all association statistically non-significant. This suggests that the apparent non-trivial association of epigenetic age acceleration with survival in glioblastoma likely reflects the confounding influence of mixed methylation subtypes. It is worth noting that, in a prior study (19), the authors reported a statistically significant positive association between age acceleration and survival in the Classic-like subtype within the TCGA cohort; however, this result was not replicated in an independent cohort. In our Cox regression analysis, which applied more rigorous covariate adjustment (age, sex, grade, and MGMT promoter status, with correct handling of variables violating the proportional hazards assumption), this finding was not confirmed. For example, using the same Horvath clock, we obtained an HR (95% CI) of 0.992 (0.980–1.004), p = 0.189. These discrepancies highlight the need for future studies in larger, independent cohorts.

The only statistically significant association that remains after dividing glioblastomas into subtypes is between telomere shortening and decreased survival in the PA-like subtype. Most cancer cells bypass telomere shortening by activating telomerase, enabling unlimited proliferation (100). This is achieved by activating the normally inactive human telomerase reverse transcriptase (hTERT) gene or increasing its expression. As with other solid tumors, gliomas exhibit telomerase activity or hTERT expression, which is absent in normal brain tissue (101, 102). Although telomerase activity and slower telomere shortening would be expected to indicate more advanced malignancy, our analysis instead found that accelerated telomere shortening was associated with decreased survival. This may reflect telomere crisis, a phenomenon documented in lymphocytic leukemia, breast cancer, and colorectal adenoma (103–105). During crisis, cell populations typically decline as proliferative potential is suppressed by cell death, making crisis an intrinsic barrier against cells that have lost p53- and pRb-dependent checkpoints. Yet this protection comes at a cost: the accompanying genomic instability increases the mutational burden and generates extensive genetic diversity subject to Darwinian selection, thereby fueling clonal evolution and malignant progression. Another explanation for the association between shorter telomeres and lower survival is the influence of patient age. In one study, age was significantly associated with telomerase status (106). The survival advantage for patients with telomerase-negative tumors was observed only in those younger than 60 years at diagnosis. Thus, the better prognosis of telomerase-negative patients was age-dependent, arguing against an independent prognostic value for telomerase-associated parameters in glioblastoma.

A similar result was obtained for oligodendrogliomas: telomere shortening was associated with decreased survival. In this tumor subtype, accelerated epigenetic aging was also consistently associated with reduced survival, a finding confirmed across nine clocks. Thus, accelerated epigenetic senescence in oligodendrogliomas can be regarded as an integral indicator of poor prognosis. This is supported by evidence that specific epigenetic alterations accumulate during oligodendroglioma progression. Studies on paired primary and recurrent tumor samples have shown that MGMT promoter methylation increases with progression (107), suggesting that more aggressive forms of these tumors are indeed characterized by dynamic methylome changes. Our results demonstrate that aging mechanisms – replicative senescence via telomere attrition and epigenetic drift – are significant determinants of aggressiveness even within the prognostically favorable group of oligodendrogliomas. This does not contradict their generally favorable outcome but rather underscores the inherent heterogeneity of this group, where epigenetic alterations can shape the prognosis for an individual patient. In essence, epigenetic clocks and telomere length can serve as integral biomarkers for risk stratification within oligodendroglioma subtypes.

Summing up, the direction and prognostic significance of epigenetic acceleration in gliomas depend fundamentally on the tumor’s molecular subtype, and no universal “greater acceleration, worse prognosis” pattern exists. This further underscores the marked heterogeneity of these tumors. When discussing accelerated epigenetic aging in glioma patients, it is important to emphasize that different epigenetic clocks estimate biological age in distinct ways. By employing multiple epigenetic clock models, our study aims not merely to document accelerated aging, but to assess which existing algorithms best correlate with clinical outcomes across different glioma subtypes. This approach avoids oversimplification and, in the future, may enable a more personalized interpretation of age-related changes in tumor tissue.

Several perspective lines of research arise. First, independent validation in larger cohorts is essential. Second, mechanistic studies employing epigenetic enzyme modulation in glioma models are required to establish a causal relationship between accelerated epigenetic aging and tumor progression. Third, longitudinal assessment of epigenetic age dynamics during therapy may open new avenues for disease monitoring. Finally, further biological deconvolution of signals from different epigenetic clocks could help determine which aspects of epigenetic dysregulation are most clinically significant across distinct glioma subtypes.

Another important aspect of gliomas, besides epigenetic aging, is inflammatory aging, since it may influence the efficacy of immunotherapy. In gliomas, inflammaging switches microglia and macrophages to a pro-tumorigenic phenotype, hyperactivates the PD-1/PD-L1 axis, and induces systemic immunosuppression (28, 108). However, a fundamental question remains: is inflammaging an independent driver of glioma progression, or does the tumor itself accelerate local and systemic aging? In glioblastoma, age appears to influence immunotherapy response in both primary and recurrent tumors, yet current clinical trial data are insufficient to fully capture this complex variable. Existing studies are extremely limited, and most share a serious flaw: immunotherapy trials overwhelmingly enroll younger patients and systematically exclude the elderly (28), significantly distorting the true spectrum of therapeutic responses. This issue is further compounded by the fact that biological age offers an additional, clinically relevant perspective. An inflammatory aging clock applied to glioblastoma showed that patients with a lower estimated inflammatory age had significantly better overall survival than those with high values (109). This finding reinforces that the biology of aging, as captured by metrics like the inflammatory clock, represents a distinct axis of the disease – independent of a patient’s chronological age – and one that likely influences the tumor’s susceptibility to immune-based interventions. Although age influences all anticancer therapies, immunotherapy may be especially vulnerable to age-related changes due to the well-documented decline of the immune system with age. Therefore, patient age – both chronological and biological – must be considered a key clinical characteristic when designing clinical trials for glioblastoma.

## 7 Conclusion

Our findings support the idea that glioma progression and aging may interact through partially shared molecular mechanisms. The biomarkers of malignancy and survival discussed in this paper also appear to be markers of aging and may serve as potential targets for future research. However, chronological aging alone cannot fully capture the molecular pathogenesis or biological behavior of gliomas. A detailed analysis of epigenetic age in these tumors revealed that its effect on survival varies by glioma subtype. This highlights that epigenetic aging acts as a subtype-specific phenomenon rather than a universal marker of malignancy. Inflammatory aging represents another important aspect in the context of glioma treatment. Assessing inflammatory age can help stratify patients for immunotherapeutic approaches by identifying those with the most pronounced inflammatory background, who may therefore be more resistant to treatment. Thus, integrating biological age data into treatment plans could improve therapeutic efficacy in glioma patients by accounting for individual patient variability. From the current perspective, personalized profiling of aging appears promising both the fundamental understanding the development of gliomas and their treatment, although the best is clearly yet to be done.

## Data Availability

Publicly available datasets were analyzed in this study. This data can be found here: DNA methylation data from the TCGA-LGG and TCGA-GBM glioma datasets are publicly available in The Cancer Genome Atlas (TCGA) repository (https://portal.gdc.cancer.gov/, accessed on 11 March 2026), while expanded clinical information can be accessed via cBioPortal (https://www.cbioportal.org/). Datasets containing frontal cortex samples from control patients are available in the Gene Expression Omnibus (GEO) repository (https://www.ncbi.nlm.nih.gov/geo/) under accession numbers GSE41826, GSE40360, GSE53162, and GSE59457.

## References

[1] Weller M Wen PY Chang SM Dirven L Lim M Monje M . Glioma. Nat Rev Dis Primer. (2024) 10:33. doi: 10.1038/s41572-024-00516-y 38724526

[2] Kinnersley B Jung J Cornish AJ Chubb D Laxton R Frangou A . Genomic landscape of diffuse glioma revealed by whole genome sequencing. Nat Commun. (2025) 16:4233. doi: 10.1038/s41467-025-59156-9 40335506 PMC12059081

[3] Louis DN Perry A Wesseling P Brat DJ Cree IA Figarella-Branger D . The 2021 WHO classification of tumors of the central nervous system: a summary. Neuro-Oncol. (2021) 23:1231–51. doi: 10.1093/neuonc/noab106 34185076 PMC8328013

[4] Pećina-Šlaus N Zottel A Škripek Ž Puljko B Dumančić F Bukovac A . In silico analysis reveals distinct changes in markers of epithelial to mesenchymal transition in glioma subtypes. Biomol BioMed. (2025) 25:2712–36. doi: 10.17305/bb.2025.12598 40679044 PMC12461275

[5] Brown NF Ottaviani D Tazare J Gregson J Kitchen N Brandner S . Survival outcomes and prognostic factors in glioblastoma. Cancers. (2022) 14:3161. doi: 10.3390/cancers14133161 35804940 PMC9265012

[6] Lee JH Lee JE Kahng JY Kim SH Park JS Yoon SJ . Human glioblastoma arises from subventricular zone cells with low-level driver mutations. Nature. (2018) 560:243–7. doi: 10.1038/s41586-018-0389-3 30069053

[7] Pećina-Šlaus N Hrašćan R . Glioma stem cells—features for new therapy design. Cancers. (2024) 16:1557. doi: 10.3390/cancers16081557 38672638 PMC11049195

[8] Arce VM González-Rendo L Porres-Ventín L González-Álvarez V Caamaño-Teixeira S Almengló C . Neural stem cells and glioblastoma stem cells: redefining concepts. Crit Rev Oncol Hematol. (2026) 220:105187. doi: 10.1016/j.critrevonc.2026.105187 41651314

[9] Neftel C Laffy J Filbin MG Hara T Shore ME Rahme GJ . An integrative model of cellular states, plasticity, and genetics for glioblastoma. Cell. (2019) 178:835–849.e21. doi: 10.1016/j.cell.2019.06.024 31327527 PMC6703186

[10] Piper K DePledge L Karsy M Cobbs C . Glioma stem cells as immunotherapeutic targets: advancements and challenges. Front Oncol. (2021) 11:615704. doi: 10.3389/fonc.2021.615704 33718170 PMC7945033

[11] De Silva MI Stringer BW Bardy C . Neuronal and tumourigenic boundaries of glioblastoma plasticity. Trends Cancer. (2023) 9:223–36. doi: 10.1016/j.trecan.2022.10.010 36460606

[12] Huang J Xie Y Sun X Zeh HJ Kang R Lotze MT . DAMPs, ageing, and cancer: the ‘DAMP hypothesis’. Ageing Res Rev. (2015) 24:3–16. doi: 10.1016/j.arr.2014.10.004 25446804 PMC4416066

[13] Jackaman C Tomay F Duong L Abdol Razak NB Pixley FJ Metharom P . Aging and cancer: the role of macrophages and neutrophils. Ageing Res Rev. (2017) 36:105–16. doi: 10.1016/j.arr.2017.03.008 28390891

[14] Bottazzi B Riboli E Mantovani A . Aging, inflammation and cancer. Semin Immunol. (2018) 40:74–82. doi: 10.1016/j.smim.2018.10.011 30409538

[15] Price M Ballard CAP Benedetti JR Kruchko C Barnholtz-Sloan JS Ostrom QT . CBTRUS statistical report: primary brain and other central nervous system tumors diagnosed in the United States in 2018–2022. Neuro-Oncol. (2025) 27:iv1–iv66. doi: 10.1093/neuonc/noaf194 41092086 PMC12527013

[16] Ceccarelli M Barthel FP Malta TM Sabedot TS Salama SR Murray BA . Molecular profiling reveals biologically discrete subsets and pathways of progression in diffuse glioma. Cell. (2016) 164:550–63. doi: 10.1016/j.cell.2015.12.028 26824661 PMC4754110

[17] Chen S Jiang Y Wang C Tong S He Y Lu W . Epigenetic clocks and gliomas: unveiling the molecular interactions between aging and tumor development. Front Mol Biosci. (2024) 11:1446428. doi: 10.3389/fmolb.2024.1446428 39130373 PMC11310061

[18] Liao P Ostrom QT Stetson L Barnholtz-Sloan JS . Models of epigenetic age capture patterns of DNA methylation in glioma associated with molecular subtype, survival, and recurrence. Neuro-Oncol. (2018) 20:942–53. doi: 10.1093/neuonc/noy003 29432558 PMC6007761

[19] Zheng C Berger NA Li L Xu R . Epigenetic age acceleration and clinical outcomes in gliomas. PloS One. (2020) 15:e0236045. doi: 10.1371/journal.pone.0236045 32692766 PMC7373289

[20] Bady P Marosi C Weller M Grønberg BH Schultz H Taphoorn MJB . DNA methylation-based age acceleration observed in IDH wild-type glioblastoma is associated with better outcome—including in elderly patients. Acta Neuropathol Commun. (2022) 10:39. doi: 10.1186/s40478-022-01344-5 35331339 PMC8944086

[21] Cakmak P Jurmeister P Divé I Zeiner PS Steinbach JP Fenton TR . DNA methylation-based analysis reveals accelerated epigenetic aging in giant cell-enriched adult-type glioblastoma. Clin Epigenet. (2024) 16:179. doi: 10.1186/s13148-024-01793-w 39663543 PMC11636044

[22] Jones KA Gilder AS Lam MS Du N Banki MA Merati A . Selective coexpression of VEGF receptor 2 in EGFRvIII-positive glioblastoma cells prevents cellular senescence and contributes to their aggressive nature. Neuro-Oncol. (2016) 18:667–78. doi: 10.1093/neuonc/nov243 26420897 PMC4827039

[23] Putavet DA De Keizer PLJ . Residual disease in glioma recurrence: a dangerous liaison with senescence. Cancers. (2021) 13:1560. doi: 10.3390/cancers13071560 33805316 PMC8038015

[24] Tarumi W Murai K Nakahata Y Masui K . The paradox of senescence in glioblastoma: SASP as an emerging cancer hallmark. Cancers. (2026) 18:550. doi: 10.3390/cancers18040550 41749804 PMC12939102

[25] Barker FG Chang SM Larson DA Sneed PK Wara WM Wilson CB . Age and radiation response in glioblastoma multiforme. Neurosurgery. (2001) 49:1288–98. doi: 10.1097/00006123-200112000-00002 11846927

[26] Malmström A Grønberg BH Marosi C Stupp R Frappaz D Schultz H . Temozolomide versus standard 6-week radiotherapy versus hypofractionated radiotherapy in patients older than 60 years with glioblastoma: the Nordic randomised, phase 3 trial. Lancet Oncol. (2012) 13:916–26. doi: 10.1016/S1470-2045(12)70265-6 22877848

[27] Ohno M Miyakita Y Takahashi M Igaki H Matsushita Y Ichimura K . Survival benefits of hypofractionated radiotherapy combined with temozolomide or temozolomide plus bevacizumab in elderly patients with glioblastoma aged ≥ 75 years. Radiat Oncol. (2019) 14:200. doi: 10.1186/s13014-019-1389-7 31718669 PMC6852964

[28] Shireman JM Ammanuel S Cheng L Distler E Tao Y Kendziorski C . Glioblastoma immunotherapy in the context of the aging immune system: a systematic review and meta-analysis. J Neuro-Oncol. (2026) 176:164. doi: 10.1007/s11060-025-05395-1 41524915 PMC12795865

[29] Leblanc H Buzharsky M Varelas X Binello E Ramirez S . Age-dependent glioma progression and functional decline in a syngeneic murine model: host vulnerabilities and opportunities for targeted intervention. Neuro-Oncol Adv. (2026) 8:vdaf222. doi: 10.1093/noajnl/vdaf222 41613043 PMC12848224

[30] Coppola D Balducci L Chen D-T Loboda A Nebozhyn M Staller A . Senescence-associated-gene signature identifies genes linked to age, prognosis, and progression of human gliomas. J Geriatr Oncol. (2014) 5:389–99. doi: 10.1016/j.jgo.2014.08.003 25220188

[31] Xiao G Zhang X Zhang X Chen Y Xia Z Cao H . Aging-related genes are potential prognostic biomarkers for patients with gliomas. Aging. (2021) 13:13239–63. doi: 10.18632/aging.203008 33946049 PMC8148480

[32] Mu J Gong J Shi M Zhang Y . Analysis and validation of aging-related genes in prognosis and immune function of glioblastoma. BMC Med Genomics. (2023) 16:109. doi: 10.1186/s12920-023-01538-3 37208656 PMC10197415

[33] Wei W Dang Y Chen G Han C Zhang S Zhu Z . Comprehensive analysis of senescence-related genes identifies prognostic clusters with distinct characteristics in glioma. Sci Rep. (2025) 15:9540. doi: 10.1038/s41598-025-93482-8 40108265 PMC11923138

[34] Vershinina O Turubanova V Krivonosov M Trukhanov A Ivanchenko M . Explainable machine learning models for glioma subtype classification and survival prediction. Cancers. (2025) 17:2614. doi: 10.3390/cancers17162614 40867242 PMC12384855

[35] Shim HS Iaconelli J Shang X Li J Lan ZD Jiang S . TERT activation targets DNA methylation and multiple aging hallmarks. Cell. (2024) 187:4030–4042.e13. doi: 10.1016/j.cell.2024.05.048 38908367 PMC11552617

[36] Krause K-H . Aging: a revisited theory based on free radicals generated by NOX family NADPH oxidases. Exp Gerontol. (2007) 42:256–62. doi: 10.1016/j.exger.2006.10.011 17126513

[37] Vendrov AE Vendrov KC Smith A Yuan J Sumida A Robidoux J . NOX4 NADPH oxidase-dependent mitochondrial oxidative stress in aging-associated cardiovascular disease. Antioxid Redox Signal. (2015) 23:1389–409. doi: 10.1089/ars.2014.6221 26054376 PMC4692134

[38] Freitas-Rodríguez S Folgueras AR López-Otín C . The role of matrix metalloproteinases in aging: tissue remodeling and beyond. Biochim Biophys Acta BBA - Mol Cell Res. (2017) 1864:2015–25. doi: 10.1016/j.bbamcr.2017.05.007 28499917

[39] Goto M Chiba J Matsuura M Iwaki-Egawa S Watanabe Y . Inflammageing assessed by MMP9 in normal Japanese individuals and the patients with Werner syndrome. Intractable Rare Dis Res. (2016) 5:103–8. doi: 10.5582/irdr.2016.01028 27195193 PMC4869575

[40] Gilbert TM Zürcher NR Catanese MC Tseng C-E Di Biase MA Lyall AE . Neuroepigenetic signatures of age and sex in the living human brain. Nat Commun. (2019) 10:2945. doi: 10.1038/s41467-019-11031-0 31270332 PMC6610136

[41] Auzmendi-Iriarte J Moreno-Cugnon L Saenz-Antoñanzas A Grassi D De Pancorbo MM Arevalo M-A . High levels of HDAC expression correlate with microglial aging. Expert Opin Ther Targets. (2022) 26:911–22. doi: 10.1080/14728222.2022.2158081 36503367

[42] Lanke V Moolamalla STR Roy D Vinod PK . Integrative analysis of hippocampus gene expression profiles identifies network alterations in aging and Alzheimer’s disease. Front Aging Neurosci. (2018) 10:153. doi: 10.3389/fnagi.2018.00153 29875655 PMC5974201

[43] Sayed N Huang Y Nguyen K Krejciova-Rajaniemi Z Grawe AP Gao T . An inflammatory aging clock (iAge) based on deep learning tracks multimorbidity, immunosenescence, frailty and cardiovascular aging. Nat Aging. (2021) 1:598–615. doi: 10.1038/s43587-021-00082-y 34888528 PMC8654267

[44] Yusipov I Kondakova E Kalyakulina A Krivonosov M Lobanova N Bacalini MG . Accelerated epigenetic aging and inflammatory/immunological profile (ipAGE) in patients with chronic kidney disease. GeroScience. (2022) 44:817–34. doi: 10.1007/s11357-022-00540-4 35237926 PMC9135940

[45] Kalyakulina A Yusipov I Kondakova E Bacalini MG Franceschi C Vedunova M . Small immunological clocks identified by deep learning and gradient boosting. Front Immunol. (2023) 14:1177611. doi: 10.3389/fimmu.2023.1177611 37691946 PMC10485620

[46] Kalyakulina A Yusipov I Trukhanov A Franceschi C Moskalev A Ivanchenko M . EpInflammAge: epigenetic-inflammatory clock for disease-associated biological aging based on deep learning. Int J Mol Sci. (2025) 26:6284. doi: 10.3390/ijms26136284 40650062 PMC12249966

[47] Larrayoz IM Ferrero H Martisova E Gil-Bea FJ Ramírez MJ Martínez A . Adrenomedullin contributes to age-related memory loss in mice and is elevated in aging human brains. Front Mol Neurosci. (2017) 10:384. doi: 10.3389/fnmol.2017.00384 29187812 PMC5694777

[48] Johnson AA Shokhirev MN Wyss-Coray T Lehallier B . Systematic review and analysis of human proteomics aging studies unveils a novel proteomic aging clock and identifies key processes that change with age. Ageing Res Rev. (2020) 60:101070. doi: 10.1016/j.arr.2020.101070 32311500

[49] Cho Y-E Latour LL Kim H Turtzo LC Olivera A Livingston WS . Older age results in differential gene expression after mild traumatic brain injury and is linked to imaging differences at acute follow-up. Front Aging Neurosci. (2016) 8. doi: 10.3389/fnagi.2016.00168 27468266 PMC4942460

[50] Sun M Brady RD Casillas-Espinosa PM Wright DK Semple BD Kim HA . Aged rats have an altered immune response and worse outcomes after traumatic brain injury. Brain Behav Immun. (2019) 80:536–50. doi: 10.1016/j.bbi.2019.04.038 31039431

[51] Cummins MJ Cresswell ET Smith DW . Intermittent fasting attenuates CNS inflammaging - rebalancing the transposonome. (2025). doi: 10.21203/rs.3.rs-6165725/v1

[52] Tian X Zhao Z Zhao J Su D He B Shi C . Transcriptomic analysis to identify genes associated with hypothalamus vulnerability in aging mice with cognitive decline. Behav Brain Res. (2024) 465:114943. doi: 10.1016/j.bbr.2024.114943 38452974

[53] Haberman RP Colantuoni C Stocker AM Schmidt AC Pedersen JT Gallagher M . Prominent hippocampal CA3 gene expression profile in neurocognitive aging. Neurobiol Aging. (2011) 32:1678–92. doi: 10.1016/j.neurobiolaging.2009.10.005 19913943 PMC2891610

[54] Butler M Pongor L Su Y-T Xi L Raffeld M Quezado M . MGMT status as a clinical biomarker in glioblastoma. Trends Cancer. (2020) 6:380–91. doi: 10.1016/j.trecan.2020.02.010 32348734 PMC7315323

[55] Barnett AE Ozair A Bamashmos AS Li H Bosler DS Yeaney G . MGMT methylation and differential survival impact by sex in glioblastoma. Cancers. (2024) 16:1374. doi: 10.3390/cancers16071374 38611052 PMC11011031

[56] Hwang T Mathios D McDonald KL Daris I Park S-H Burger PC . Integrative analysis of DNA methylation suggests down-regulation of oncogenic pathways and reduced somatic mutation rates in survival outliers of glioblastoma. Acta Neuropathol Commun. (2019) 7:88. doi: 10.1186/s40478-019-0744-0 31159876 PMC6545689

[57] Horvath S . DNA methylation age of human tissues and cell types. Genome Biol. (2013) 14:3156. doi: 10.1186/gb-2013-14-10-r115 24138928 PMC4015143

[58] Hannum G Guinney J Zhao L Zhang L Hughes G Sadda S . Genome-wide methylation profiles reveal quantitative views of human aging rates. Mol Cell. (2013) 49:359–67. doi: 10.1016/j.molcel.2012.10.016 23177740 PMC3780611

[59] Higgins-Chen AT Thrush KL Wang Y Minteer CJ Kuo P-L Wang M . A computational solution for bolstering reliability of epigenetic clocks: implications for clinical trials and longitudinal tracking. Nat Aging. (2022) 2:644–61. doi: 10.1038/s43587-022-00248-2 36277076 PMC9586209

[60] Warner B Ratner E Datta A Lendasse A . A systematic review of phenotypic and epigenetic clocks used for aging and mortality quantification in humans. Aging. (2024) 16:12414–27. doi: 10.18632/aging.206098 39215995 PMC11424583

[61] Oblak L Van Der Zaag J Higgins-Chen AT Levine ME Boks MP . A systematic review of biological, social and environmental factors associated with epigenetic clock acceleration. Ageing Res Rev. (2021) 69:101348. doi: 10.1016/j.arr.2021.101348 33930583

[62] Guintivano J Aryee MJ Kaminsky ZA . A cell epigenotype specific model for the correction of brain cellular heterogeneity bias and its application to age, brain region and major depression. Epigenetics. (2013) 8:290–302. doi: 10.4161/epi.23924 23426267 PMC3669121

[63] Huynh JL Garg P Thin TH Yoo S Dutta R Trapp BD . Epigenome-wide differences in pathology-free regions of multiple sclerosis–affected brains. Nat Neurosci. (2014) 17:121–30. doi: 10.1038/nn.3588 24270187 PMC3934491

[64] Ladd-Acosta C Hansen KD Briem E Fallin MD Kaufmann WE Feinberg AP . Common DNA methylation alterations in multiple brain regions in autism. Mol Psychiatry. (2014) 19:862–71. doi: 10.1038/mp.2013.114 23999529 PMC4184909

[65] Horvath S Levine AJ . HIV-1 infection accelerates age according to the epigenetic clock. J Infect Dis. (2015) 212:1563–73. doi: 10.1093/infdis/jiv277 25969563 PMC4621253

[66] De Lima Camillo LP Lapierre LR Singh R . A pan-tissue DNA-methylation epigenetic clock based on deep learning. NPJ Aging. (2022) 8:4. doi: 10.1038/s41514-022-00085-y 37880705

[67] Horvath S Oshima J Martin GM Lu AT Quach A Cohen H . Epigenetic clock for skin and blood cells applied to Hutchinson Gilford Progeria Syndrome and ex vivo studies. Aging. (2018) 10:1758–75. doi: 10.18632/aging.101508 30048243 PMC6075434

[68] Shireby GL Davies JP Francis PT Burrage J Walker EM Neilson GWA . Recalibrating the epigenetic clock: implications for assessing biological age in the human cortex. Brain. (2020) 143:3763–75. doi: 10.1093/brain/awaa334 33300551 PMC7805794

[69] Lu AT Quach A Wilson JG Reiner AP Aviv A Raj K . DNA methylation GrimAge strongly predicts lifespan and healthspan. Aging. (2019) 11:303–27. doi: 10.18632/aging.101684 30669119 PMC6366976

[70] Lu AT Binder AM Zhang J Yan Q Reiner AP Cox SR . DNA methylation GrimAge version 2. Aging. (2022) 14:9484–549. doi: 10.18632/aging.204434 36516495 PMC9792204

[71] Levine ME Lu AT Quach A Chen BH Assimes TL Bandinelli S . An epigenetic biomarker of aging for lifespan and healthspan. Aging. (2018) 10:573–91. doi: 10.18632/aging.101414 29676998 PMC5940111

[72] Lin Q Weidner CI Costa IG Marioni RE Ferreira MRP Deary IJ . DNA methylation levels at individual age-associated CpG sites can be indicative for life expectancy. Aging. (2016) 8:394–401. doi: 10.18632/aging.100908 26928272 PMC4789590

[73] Ying K Liu H Tarkhov AE Sadler MC Lu AT Moqri M . Causality-enriched epigenetic age uncouples damage and adaptation. Nat Aging. (2024) 4:231–46. doi: 10.1038/s43587-023-00557-0 38243142 PMC11070280

[74] Tong H Dwaraka VB Chen Q Luo Q Lasky-Su JA Smith R . Quantifying the stochastic component of epigenetic aging. Nat Aging. (2024) 4:886–901. doi: 10.1038/s43587-024-00600-8 38724732 PMC11186785

[75] Yang Z Wong A Kuh D Paul DS Rakyan VK Leslie RD . Correlation of an epigenetic mitotic clock with cancer risk. Genome Biol. (2016) 17:205. doi: 10.1186/s13059-016-1064-3 27716309 PMC5046977

[76] Zhu T Tong H Du Z Beck S Teschendorff AE . An improved epigenetic counter to track mitotic age in normal and precancerous tissues. Nat Commun. (2024) 15:4211. doi: 10.1038/s41467-024-48649-8 38760334 PMC11101651

[77] Endicott JL Nolte PA Shen H Laird PW . Cell division drives DNA methylation loss in late-replicating domains in primary human cells. Nat Commun. (2022) 13:6659. doi: 10.1038/s41467-022-34268-8 36347867 PMC9643452

[78] Lu AT Seeboth A Tsai P-C Sun D Quach A Reiner AP . DNA methylation-based estimator of telomere length. Aging. (2019) 11:5895–923. doi: 10.18632/aging.102173 31422385 PMC6738410

[79] De Lima Camillo LP . pyaging: a Python-based compendium of GPU-optimized aging clocks. Bioinformatics. (2024) 40:btae200. doi: 10.1093/bioinformatics/btae200 38603598 PMC11058068

[80] Stevenson AJ McCartney DL Gadd DA Shireby G Hillary RF King D . A comparison of blood and brain‐derived ageing and inflammation‐related DNA methylation signatures and their association with microglial burdens. Eur J Neurosci. (2022) 56:5637–49. doi: 10.1111/ejn.15661 35362642 PMC9525452

[81] Tan C Wei Y Ding X Han C Sun Z Wang C . Cell senescence-associated genes predict the Malignant characteristics of glioblastoma. Cancer Cell Int. (2022) 22:411. doi: 10.1186/s12935-022-02834-1 36527013 PMC9758946

[82] Liu Y Feng Y Cheng L Xu Y Wu A Cheng P . Profiling with senescence-associated secretory phenotype score identifies GDC-0879 as a small molecule sensitizing glioblastoma to anti-PD1. Cell Death Dis. (2025) 16:602. doi: 10.1038/s41419-025-07915-3 40781221 PMC12334699

[83] Salam R Saliou A Bielle F Bertrand M Antoniewski C Carpentier C . Cellular senescence in Malignant cells promotes tumor progression in mouse and patient glioblastoma. Nat Commun. (2023) 14:441. doi: 10.1038/s41467-023-36124-9 36707509 PMC9883514

[84] Al Shboul S Alrjoub M Al Karsaneh OA Surakhy M Alhesa A El-Sadoni M . Integrated biomarker mapping reveals differential expression of senescence profiles in IDH-wild-type glioblastoma recurrent versus primary tumors. Virchows Arch. (2026). doi: 10.1007/s00428-026-04541-y 42089920

[85] Kumari R Jat P . Mechanisms of cellular senescence: Cell cycle arrest and senescence associated secretory phenotype. Front Cell Dev Biol. (2021) 9:645593. doi: 10.3389/fcell.2021.645593 33855023 PMC8039141

[86] Liu Y Lomeli I Kron SJ . Therapy-induced cellular senescence: Potentiating tumor elimination or driving cancer resistance and recurrence? Cells. (2024) 13:1281. doi: 10.3390/cells13151281 39120312 PMC11312217

[87] Alessio N Aprile D Squillaro T Di Bernardo G Finicelli M Melone MA . The senescence-associated secretory phenotype (SASP) from mesenchymal stromal cells impairs growth of immortalized prostate cells but has no effect on metastatic prostatic cancer cells. Aging. (2019) 11:5817–28. doi: 10.18632/aging.102172 31412320 PMC6710033

[88] Ohtani N . The roles and mechanisms of senescence-associated secretory phenotype (SASP): Can it be controlled by senolysis? Inflammation Regener. (2022) 42:11. doi: 10.1186/s41232-022-00197-8 35365245 PMC8976373

[89] Cao L Li K Li Q Tong Q Wang Y Huang L . The controversial role of senescence-associated secretory phenotype (SASP) in cancer therapy. Mol Cancer. (2025) 24:283. doi: 10.1186/s12943-025-02475-8 41204284 PMC12595836

[90] Bae WH Maraka S Daher A . Challenges and advances in glioblastoma targeted therapy: The promise of drug repurposing and biomarker exploration. Front Oncol. (2024) 14:1441460. doi: 10.3389/fonc.2024.1441460 39439947 PMC11493774

[91] Chiariello M Inzalaco G Barone V Gherardini L . Overcoming challenges in glioblastoma treatment: Targeting infiltrating cancer cells and harnessing the tumor microenvironment. Front Cell Neurosci. (2023) 17:1327621. doi: 10.3389/fncel.2023.1327621 38188666 PMC10767996

[92] Liu Y Zhou F Ali H Lathia JD Chen P . Immunotherapy for glioblastoma: Current state, challenges, and future perspectives. Cell Mol Immunol. (2024) 21:1354–75. doi: 10.1038/s41423-024-01226-x 39406966 PMC11607068

[93] Hsueh B Steuart SJ Odukoya AO Prager BC Kim YJ Hill CM . Immunotherapy and targeted therapy for high grade gliomas: Current and future directions. J Neuro-Oncol. (2026) 176:112. doi: 10.1007/s11060-025-05315-3 41366148

[94] Massara M Persico P Bonavita O Mollica Poeta V Locati M Simonelli M . Neutrophils in gliomas. Front Immunol. (2017) 8:1349. doi: 10.3389/fimmu.2017.01349 29123517 PMC5662581

[95] Nicolaou N Andreou MS Neophytou CM Papageorgis P . Emerging insights into the immunosuppressive tumor microenvironment and its implications for glioblastoma immunotherapy. Front Immunol. (2025) 16:1665742. doi: 10.3389/fimmu.2025.1665742 41208963 PMC12592078

[96] Chen X Wang J Liu S Han Q Zhang H Wu J . Research landscape of glioma and inflammation over the past two decades. Front Immunol. (2025) 16:1605346. doi: 10.3389/fimmu.2025.1605346 40881678 PMC12381528

[97] Xu S Tang L Li X Fan F Liu Z . Immunotherapy for glioma: Current management and future application. Cancer Lett. (2020) 476:1–12. doi: 10.1016/j.canlet.2020.02.002 32044356

[98] Jabri A Mhannayeh A Taftafa B Alsharif M Sibai D Alsharif R . Recent advances in immunotherapy for gliomas: Overcoming barriers and advancing precision strategies. Front Immunol. (2026) 16:1690464. doi: 10.3389/fimmu.2025.1690464 41583467 PMC12823976

[99] Turcan S Rohle D Goenka A Walsh LA Fang F Yilmaz E . IDH1 mutation is sufficient to establish the glioma hypermethylator phenotype. Nature. (2012) 483:479–83. doi: 10.1038/nature10866 22343889 PMC3351699

[100] Shay JW Bacchetti S . A survey of telomerase activity in human cancer. Eur J Cancer. (1997) 33:787–91. doi: 10.1016/S0959-8049(97)00062-2 9282118

[101] Shervington A Patel R Lu C Cruickshanks N Lea R Roberts G . Telomerase subunits expression variation between biopsy samples and cell lines derived from Malignant glioma. Brain Res. (2007) 1134:45–52. doi: 10.1016/j.brainres.2006.11.093 17196947

[102] Shervington A Patel R . Differential hTERT mRNA processing between young and older glioma patients. FEBS Lett. (2008) 582:1707–10. doi: 10.1016/j.febslet.2008.04.027 18435920

[103] Maciejowski J De Lange T . Telomeres in cancer: tumour suppression and genome instability. Nat Rev Mol Cell Biol. (2017) 18:175–86. doi: 10.1038/nrm.2016.171 28096526 PMC5589191

[104] Nassour J Karlseder J . Telomere crisis shapes cancer evolution. Cold Spring Harb Perspect Biol. (2026) 18:a041688. doi: 10.1101/cshperspect.a041688 40789644 PMC12403157

[105] Dewhurst SM Yao X Rosiene J Tian H Behr J Bosco N . Structural variant evolution after telomere crisis. Nat Commun. (2021) 12:2093. doi: 10.1038/s41467-021-21933-7 33828097 PMC8027843

[106] Lötsch D Ghanim B Laaber M Wurm G Weis S Lenz S . Prognostic significance of telomerase-associated parameters in glioblastoma: effect of patient age. Neuro-Oncol. (2013) 15:423–32. doi: 10.1093/neuonc/nos329 23393205 PMC3607268

[107] Lavon I Zrihan D Zelikovitch B Fellig Y Fuchs D Soffer D . Longitudinal assessment of genetic and epigenetic markers in oligodendrogliomas. Clin Cancer Res. (2007) 13:1429–37. doi: 10.1158/1078-0432.CCR-06-2050 17332285

[108] Ladomersky E Scholtens DM Kocherginsky M Hibler EA Bartom ET Otto-Meyer S . The coincidence between increasing age, immunosuppression, and the incidence of patients with glioblastoma. Front Pharmacol. (2019) 10:200. doi: 10.3389/fphar.2019.00200 30971917 PMC6446059

[109] Zhu L Wang F Huang J Wang H Wang G Jiang J . Inflammatory aging clock: a cancer clock to characterize the patients’ subtypes and predict the overall survival in glioblastoma. Front Genet. (2022) 13:925469. doi: 10.3389/fgene.2022.925469 36035122 PMC9402943

